# Assessment of psychometric properties of the Korean SF-12 v2 in the general population

**DOI:** 10.1186/1471-2458-14-1086

**Published:** 2014-10-18

**Authors:** Seon-Ha Kim, Min-Woo Jo, Jeonghoon Ahn, Minsu Ock, Sangjin Shin, Jooyeon Park

**Affiliations:** Department of Nursing, Dankook University, 119 Dandae-ro, Dongnam-gu, Cheonan-si, Chungnam, 330-714 South Korea; Department of Preventive Medicine, University of Ulsan College of Medicine, 86, Asanbyeongwon-gil, Songpa-gu, Seoul, 138-736 South Korea; Office of Health Services Research, National Evidence-based Healthcare Collaborating Agency, Namasan Square 7F 173, Toegye-ro, Jung-gu, Seoul, 100-705 South Korea

**Keywords:** SF-12, Quality of life, Korea, Validity, Population

## Abstract

**Background:**

The psychometric properties of the Korean Short Form-12 Health Survey, version 2 (SF-12 v2) have not been assessed in the general population. Therefore, the aim of our study was to evaluate the psychometric properties of the Korean version of the SF-12 v2 in the general population and to provide SF-12 v2 domain scores according to the general characteristics of the study population.

**Methods:**

A total of 1,000 participants from the general Korean population were recruited using a multistage quota sampling method. Psychometric properties were evaluated by descriptive statistics, validity, reliability, and exploratory factor analysis.

**Results:**

Item convergent and discriminant validity met the criteria established by the instrument developer. In the known-group comparison, male gender, age <60 years, high educational status, and absence of any comorbidity were significantly associated with high scale scores. The reliability of all SF-12 v2 items was 0.88.

**Conclusions:**

The findings of this study generally support the idea that the Korean SF-12 v2 is a feasible, valid, and reliable instrument for assessing health-related quality of life in the general population. The SF-12 v2 seems to be a viable alternative health-related quality of life instrument for the Korean population.

## Background

Interest in health-related quality of life (HRQoL) issues has increased in recent decades, and the number of citations for “quality of life” in the medical literature has increased significantly. HRQoL instruments are essential for evaluating HRQoL as an outcome measure of community- or hospital-based interventions [1]. The Short Form-36 Health Survey, version 2 (SF-36 v2) is one of the most popular generic worldwide instruments for evaluating HRQoL. The SF-12 v2 is a shorter version of the SF-36 v2 that uses only 12 questions. Because the SF-12 v2 is brief and measures various aspects of health status, it has become the instrument of choice in population health surveys and in clinical studies that combine it with disease-specific instruments [2, 3]. Several studies have reported the validity and reliability of the SF-12 as a measure of HRQoL in a range of medical conditions, as well as in the general population [4–8]. Although the psychometric properties of the Korean SF-36 v2 have been evaluated in the general population [9, 10], a similar evaluation of the psychometric properties of the Korean SF-12 v2 is yet to be performed.

In addition, there is some evidence suggesting cultural differences in the item interpretation of HRQoL instruments [11, 12]. Therefore, assessing the feasibility and understanding the psychometric properties of the instruments should precede their application in research when instruments developed in other countries are adapted to the Korean population. Therefore, the aim of our current study was to evaluate the psychometric properties of the Korean version of the SF-12 v2 in the general population and to provide SF-12 v2 domain scores according to the general characteristics of the study population.

## Methods

### Study design

This study was conducted using individual face-to-face interviews. The survey was performed from August 2013 to November 2013 by 27 trained interviewers. Respondents were asked to complete the Korean version of the SF-12 v2 for HRQoL. Data on demographic factors (i.e. age, sex, level of education, and occupation) and health-related factors (i.e. current disease, outpatient visits in the past 2 weeks, and hospitalization in the past year) were also collected.

### Setting and samples

Out of the 3,206 households that were contacted for interviews, 1,000 successful interviews were conducted (31.2%). The target population included individuals aged 19 years or older living in Korea (except for Jeju Island) who consented to participate in the survey. Sampling was performed using a multistage stratified quota method. Sample quota were assigned to each of the 15 Korean regions according to the population structure (gender, 10-year age group, and level of education [12 years or less vs. more than 12 years]), as defined by the resident registration data of the Ministry of Administration and Security of South Korea in June 2013.

### Ethical considerations

This study was approved by the Institutional Review Board of the National Evidence-based healthcare Collaborating Agency (approval number: NECA IRB13-002), and all of the participants provided written informed consent.

### Measurements

Our present study used the Korean SF-12 v2. The SF-12 v2 is a multipurpose, short form, health survey that includes 12 items taken directly from the SF-36 v2. The SF-12 v2 yields eight scale scores (physical functioning [PF], role-physical [RP], bodily pain [BP], general health [GH], vitality [VT], social functioning [SF], role-emotional [RE], and mental health [MH]). Four scale scores (PF, RP, RE, and MH) are calculated using two items each, whereas the remaining scales (BP, GH, VT, and SF) are represented by a single item [13]. Several worded items were recoded so that higher scores indicate a better condition. Scale scores were transformed into the 0 to 100 range according to the scoring manual [14]. The 12 items are used to derive two summary measures (i.e. physical component summary [PCS] and mental component summary [MCS]) [15].

### Data analysis

The SF-12 v2 was assessed according to the data quality indicator recommended by its developer [13]. The assessment included completeness of the data, based on the percentage of the total number of items with a valid item response, as well as on the percentage of responses outside the range. In addition, convergent validity was tested to determine whether items were expected to represent the PCS or the MCS. When all of the hypothesized item-component correlations were 0.30 or greater, convergent validity was considered to be acceptable. It was hypothesized that the PCS is related to the PF, RP, GH, and BP items, and the MCS is related to the MH, RE, VT, and SF items. Finally, discriminant validity was assessed to determine whether an item more highly correlates with its hypothesized component summary measure score than with the alternative component summary measure score. When all of the hypothesized item-component correlations were significantly higher than the alternative item-component correlations, item discriminant validity was considered to be satisfactory. In addition, the percentages of respondents who achieved either the highest score (ceiling) or the lowest score (floor) were calculated because large ceiling and floor effects may limit the responsiveness of the SF-12 v2 [9, 13].

To assess construct validity, SF-12 v2 scale scores were calculated in terms of sociodemographic and health-related factors. It was expected that the SF-12 v2 scale scores would be lower in women, older persons, poorly educated persons, the unemployed, those suffering from any disease, and recent health service users [11, 16–19]. Comparison of differences in scale scores between groups was performed using the student’s *t*-test or analysis of variance with post hoc Tukey’s test.

The summary measure, internal reliability, was analyzed with Cronbach’s alpha. When Cronbach’s alpha was ≥0.7, the reliability was considered to be acceptable [20]. To test whether the Korean SF-12 v2 produced the hypothesized structure of the original survey, exploratory item level factor analysis was performed using principal component analysis with varimax rotation. Factor loadings ≥0.4 were considered to be significant [21]. All statistical analyses were conducted using SAS (version 9.1; SAS Institute Inc., Cary, NC).

## Results

The mean age of the participants was 45.0 years (standard deviation [SD], 14.3) and 50.1% of the participants were women. A total of 126 participants (12.6%) reported a current disease, and most of the participants were employed or self-employed (Table 1). The completeness of the data was 100%, and there were no out-of-range values. SF-12 v2 item descriptive statistics are presented in Table 2. The ceiling effect was considerably higher for the PF, RP, BP, SF, and RE items, whereas only 23 participants (2.3%) responded in the upper end of the scale for all items. The floor effect was <2% for the majority of items.

**Table 1:** **General characteristics of the study respondents**

Characteristics	Survey data		National registry (2013)
	N	(%)	%
Gender			
Male	499	(49.9)	49.5
Female	501	(50.1)	50.5
Age group			
19–29 years	183	(18.3)	17.9
30–39 years	199	(19.9)	19.9
40–49 years	209	(20.9)	21.7
50–59 years	197	(19.7)	19.5
60 years or more	212	(21.2)	21.0
Level of education			National Census (2010)
Lower than university degree	746	(74.6)	70.1
University degree or higher	254	(25.4)	29.9
Occupation			
Employed/business person	700	(70.0)	
Housewife	215	(21.5)	
Student	53	(5.3)	
Unemployed	32	(3.2)	
Marital status			
Married	711	(71.0)	
Widowed/Divorced	53	(5.3)	
Unmarried	236	(23.6)	
Current disease			
Yes	126	(12.6)	
No	874	(87.4)	
Outpatient visit in the past 2 weeks			
Yes	145	(14.5)	
No	855	(85.5)	
Hospitalization in the past year			
Yes	45	(4.5)	
No	955	(95.5)

**Table 2:** **SF-12 v2 items and descriptive statistics (n =1000)**

Item description (scale)	Mean	SD	Item response category frequency (%)				
			1	2	3	4	5
Moderate activities (PF)	2.85	0.40	1.6	11.6	86.8	NA	NA
Climb several flights of stairs (PF)	2.81	0.45	2.4	14.5	83.1	NA	NA
Accomplished less, physical (RP)	4.53	0.84	0.8	3.6	7.4	18.1	70.1
Limited in kind of work (RP)	4.72	0.69	0.4	2.3	4.6	10.4	82.3
Pain-interference (BP)*	4.70	0.68	0.7	1.2	5.1	13.4	79.6
Health in general (GH)*	3.73	0.81	0.6	9.4	46.9	36.2	6.9
Energy (VT)*	3.71	0.90	2.1	8.4	21.6	52.4	15.5
Social time (SF)	4.76	0.61	0.3	1.1	4.1	11.5	83.0
Accomplished less, emotional (RE)	4.61	0.73	0.3	2.2	6.5	18.2	72.8
Did work less carefully (RE)	4.67	0.66	0.0	1.6	6.0	16.6	75.8
Calm and peaceful (MH)*	3.92	0.76	1.4	5.0	10.1	67.7	15.8
Downhearted and blue (MH)	4.38	0.81	0.5	2.6	10.0	32.3	54.6

NA: Not applicable because these two items have only three response options.

*Item recoded; hence, higher scores indicate a better condition.

The Spearman correlation coefficients for the SF-12 v2 items and their component summaries are shown in Table 3. All of the items were correlated with their hypothesized measures by ≥0.30. Each item and its hypothesized component demonstrated a correlation between 0.59–0.78. In terms of discriminant validity, all of the items were more highly correlated with their hypothesized components than with the alternative components.

**Table 3:** **Correlations between SF-12 v2 items and component summaries (n =1,000)**

Item description (scale)	PCS	MCS
Moderate activities (PF)	0.77	0.16
Climb several flights of stairs (PF)	0.78	0.18
Accomplished less, physical (RP)	0.59	0.45
Limited in kind of work (RP)	0.62	0.41
Pain-interference (BP)	0.70	0.27
Health in general (GH)	0.68	0.38
Energy (VT)	0.36	0.70
Social time (SF)	0.46	0.61
Accomplished less, emotional (RE)	0.36	0.64
Did work less carefully (RE)	0.35	0.67
Calm and peaceful (MH)	0.09	0.68
Downhearted and blue (MH)	0.14	0.65

The scale scores of the Korean SF-12 v2 according to the sociodemographic and health-related variables are shown in Table 4. Significant differences were observed in SF-12 v2 scale scores. As expected, the scale scores of women were significantly lower than those of men in all scales except for the SF and RE scales. The oldest age group (≥70 years) demonstrated a significantly lower value than the other age groups on most of the scales except for the MH scale when the post hoc Tukey’s comparison was applied. Highly educated people tended to report higher values than poorly educated people on all scales. People suffering from disease and those who recently used the hospital service demonstrated significantly lower scores than the other participants on most of the scales. Scale scores according to gender and age group are presented in Table 5.

**Table 4:** **SF-12 v2 scale scores according to general characteristics and health-related factors (n =1,000)**

	PF		RP		BP		GH		VT		SF		RE		MH	
Variables	Mean	SD	Mean	SD	Mean	SD	Mean	SD	Mean	SD	Mean	SD	Mean	SD	Mean	SD
Gender																
Male	93.0	18.4	92.4	16.9	94.0	15.5	70.4	19.8	70.7	22.2	**94.6** ^*^	15.3	**91.9** ^*^	15.9	80.1	15.0
Female	89.9	21.0	88.9	18.4	91.0	18.4	65.9	20.3	64.7	22.5	**93.3** ^*^	15.0	**90.0** ^*^	16.6	77.2	15.6
Age group																
19–29 years	97.0	11.9	93.6	14.3	96.3	11.3	80.4	16.0	77.3	19.9	96.2	12.3	92.8	14.7	**80.8** ^*^	14.8
30–39 years	95.1	15.2	93.2	14.2	94.0	16.5	74.0	18.3	70.4	20.7	94.6	14.6	91.3	15.7	**79.2** ^*^	14.8
40–49 years	96.5	11.1	92.7	16.4	94.3	15.2	67.8	18.4	68.7	21.5	95.8	12.2	92.5	14.5	**77.3** ^*^	16.0
50–59 years	92.4	17.8	91.1	18.0	91.5	17.9	64.3	17.7	66.1	20.6	93.5	15.8	91.2	16.8	**79.4** ^*^	15.3
60–69 years	79.2	27.9	84.9	19.8	88.3	20.3	57.0	20.9	57.8	24.9	90.7	18.7	88.5	17.5	**76.7** ^*^	15.5
70 years or older	65.4	34.0	70.2	31.8	77.9	24.8	49.8	22.4	54.8	23.5	84.6	20.1	78.8	25.2	**79.3** ^*^	17.3
Level of education (years)																
6 and below	62.5	32.2	69.5	27.9	76.6	25.5	48.9	23.0	45.3	25.1	81.8	20.5	80.7	23.3	72.7	16.8
7 to 9	77.9	28.7	86.4	18.0	86.6	21.2	55.3	21.5	55.8	23.2	90.3	18.7	88.9	16.1	75.7	16.0
10 to 12	93.8	16.0	91.8	16.4	93.9	15.3	69.7	19.3	69.1	21.7	95.1	13.6	91.7	15.7	78.8	15.7
13 or more	96.5	13.2	93.3	15.5	94.5	15.5	73.1	17.0	73.0	19.7	94.8	14.9	91.9	15.5	80.7	13.5
Occupation																
Employed/self-employed	94.1	16.3	92.5	16.2	93.9	15.5	69.9	18.9	69.5	21.4	94.9	14.3	92.0	15.4	79.6	15.0
Unemployed	70.3	30.1	82.0	22.0	84.4	24.4	52.7	24.4	57.0	24.0	88.3	17.9	83.2	19.5	76.2	14.7
Student	95.8	12.7	92.7	15.6	98.1	8.3	81.6	16.8	76.9	22.9	96.2	13.3	93.2	14.0	79.7	15.2
Housewife	84.9	25.7	85.4	20.8	87.9	20.8	61.6	21.3	61.3	24.0	91.2	17.4	88.1	18.6	75.7	16.3
Current disease																
No	94.4	15.4	92.7	15.0	94.3	15.4	71.0	17.9	70.0	21.0	95.4	13.4	92.1	15.2	79.8	14.7
Yes	71.0	31.5	76.3	26.6	80.2	22.7	48.8	24.4	52.0	26.4	83.7	21.5	83.2	21.2	70.6	17.6
Outpatient visit (in the past 2 weeks)																
No	92.1	18.7	91.1	17.2	93.3	15.9	68.7	19.7	68.3	22.2	94.4	14.5	**91.3** ^*^	15.8	79.0	15.0
Yes	77.2	32.8	80.8	25.4	76.1	29.7	56.7	25.6	55.6	26.6	84.4	23.4	**84.2** ^*^	23.6	71.7	20.2
Hospitalization (in the past year)																
No	94.1	16.1	92.6	15.7	94.5	14.8	71.0	18.0	70.2	20.8	95.5	13.4	92.3	14.9	80.2	14.3
Yes	76.2	30.1	79.1	23.8	80.5	23.7	51.3	23.7	52.9	26.4	84.7	20.9	82.8	21.3	69.5	18.2

*Bold indicates non-significant differences (P > 0.05) according to the Student’s *t*-test or analysis of variance.

**Table 5:** **Scale scores and component summary scores according to gender and age group (n =1,000)**

	PF		RP		BP		GH		VT		SF		RE		MH		PCS		MCS	
Age group (years)	Mean	SD	Mean	SD	Mean	SD	Mean	SD	Mean	SD	Mean	SD	Mean	SD	Mean	SD	Mean	SD	Mean	SD
Male																				
19–29	96.9	11.6	93.8	15.5	95.9	12.8	84.3	15.1	82.0	19.7	95.9	13.8	93.0	15.6	82.5	15.3	55.5	4.2	55.5	6.9
30–39	94.8	16.5	92.1	16.1	95.2	14.7	75.2	18.4	71.7	21.7	93.8	16.2	90.7	16.4	79.6	15.2	54.3	5.6	53.5	7.3
40–49	97.7	10.0	94.4	16.9	94.2	17.0	67.7	19.6	71.1	20.0	97.2	9.9	94.0	13.7	78.4	16.5	53.9	4.0	53.9	6.6
50–59	92.7	19.2	91.9	17.6	92.2	17.5	65.7	15.5	66.1	22.4	92.7	17.4	90.6	18.2	79.0	15.8	52.6	5.2	53.1	7.8
60–69	83.3	24.7	90.5	16.8	93.6	13.0	59.8	19.9	62.8	23.4	93.3	19.2	92.0	14.5	80.6	11.2	50.3	6.0	54.3	6.2
70 or older	75.0	36.6	82.5	25.4	86.7	22.9	51.7	21.3	60.0	20.7	91.7	12.2	86.7	20.3	85.8	10.4	46.1	9.5	55.4	5.3
Female																				
19–29	97.1	12.4	93.5	12.8	96.8	9.2	76.0	15.9	72.1	18.9	96.5	10.3	92.4	13.7	78.9	14.1	55.0	3.4	53.7	5.8
30–39	95.5	13.7	94.3	11.7	92.6	18.3	72.6	18.2	68.9	19.6	95.5	12.7	92.0	14.9	78.7	14.3	54.0	4.6	53.5	6.4
40–49	95.3	12.1	90.8	15.8	94.3	13.2	67.8	17.1	66.1	22.8	94.3	14.1	90.8	15.1	76.2	15.4	53.6	4.1	52.4	6.5
50–59	92.1	16.6	90.3	18.5	90.8	18.3	63.0	19.5	66.1	18.9	94.3	14.1	91.7	15.3	79.7	14.9	51.7	6.5	53.8	6.1
60–69	76.2	29.7	80.9	20.9	84.5	23.6	55.0	21.5	54.2	25.5	88.9	18.2	86.0	19.1	73.8	17.5	47.4	8.5	51.6	8.0
70 or older	52.3	26.1	53.4	33.1	65.9	23.1	47.3	24.7	47.7	26.1	75.0	25.0	68.2	28.2	70.5	21.1	39.0	9.3	49.1	10.5

Internal consistency reliabilities were 0.84, 0.83, and 0.85 in the PF, RP, and RE domains, respectively, whereas the reliability was 0.37 in the MH domain. The reliability of all SF-12 v2 items was 0.88. Cronbach’s alpha for the PF, RP, GH, and BP items was 0.83, and that for the MH, RE, VT, and SF items was 0.79. Item factor analysis demonstrated the presence of three factors that accounted for 65.1% of the variance. The results are presented in Table 6. The PF, BP, and GH items loaded onto the physical health concept (factor 1) and the VT, MH, and GH items separately loaded onto the psychological health concept (factor 3). The SF, RP, and RE items loaded onto factor 2.

**Table 6:** **Factor loadings of the SF-12 v2 items after varimax rotation**

Item description (scale)	Factor 1	Factor 2	Factor 3
Moderate activities (PF)	**0.84** ^*^	0.25	0.06
Climb several flights of stairs (PF)	**0.85** ^*^	0.20	0.12
Accomplished less, physical (RP)	0.29	**0.78** ^*^	0.14
Limited in kind of work (RP)	0.36	**0.76** ^*^	0.10
Pain-interference (BP)	**0.53** ^*^	0.38	0.15
Health in general (GH)	**0.57** ^*^	0.10	**0.52** ^*^
Energy (VT)	0.22	0.15	**0.81** ^*^
Social time (SF)	0.33	**0.55** ^*^	0.33
Accomplished less, emotional (RE)	0.11	**0.85** ^*^	0.20
Did work less carefully (RE)	0.13	**0.83** ^*^	0.24
Calm and peaceful (MH)	−0.02	0.15	**0.79** ^*^
Downhearted and blue (MH)	0.14	0.30	**0.47** ^*^

*Bold indicates factor loadings ≥0.4.

## Discussion

Quality of life is a critical component of healthcare. Many HRQoL outcome measures have been used in clinical and health economics research. Prior to the application of HRQoL instruments, evidence on the psychometric properties of each instrument should be considered. Our study assessed the data quality and psychometric properties of the Korean version of the SF-12 v2 in a general population sample. The rate of missing data was zero, and the quality criteria recommended by the developer of the instrument were satisfied in our study. All of the correlations between the items and their hypothesized components were >0.3, and all of the items were more highly correlated with their own hypothesized components than with other competing components. Generally, the item scores in our sample were higher than those in other countries. Korean people seem to evaluate themselves as healthy compared to people from other countries. Differences in the SF-12 v2 scale scores in terms of sex, age, educational level, health status, and use of health services showed evidence of construct validity.

Psychometric properties of the SF-12 have been demonstrated in the general population of various countries, including USA [4, 22], Israel [5], Sweden [7], Greek [8], Hong Kong [19], and so on. Psychometric properties of the SF-12 v2 in the Americans and Chinese adolescents have been presented [6, 23]. In terms of convergent and discriminant validity, all of the hypothesized item-component correlations were 0.30 or greater, and hypothesized item-component correlations were significantly higher than the alternative item-component correlations in previous publications [17], but, the study by Jakobsson et al. showed that item-component correlations argued against the suggested structure in a general elderly population (aged 75+) [7]. Scale and component score was lower in older persons, poorly educated persons, the unemployed, those suffering from any disease, and recent health service users [8, 11, 16–19]. Cheak-Zamora et al. showed high test–retest reliability of PCS (ICC = .78) and moderate reliability of MCS (ICC = .60) [6]. Factor analysis yielded two factors and hypothesized item included the same factor in some of the countries [4, 8, 17]. However, the study performed in Israel revealed three factors and physical role loaded as a separate factor [5], and the results of the study by Jakobsson et al. failed to support a two-dimensional item structure among the elderly population [6].

This study demonstrated the psychometric properties of the Korean version of SF-12 v2. The vitality (a lot of energy) and MH (calm and peaceful, and downhearted and blue) items in the Korean population scored lower than those in Greek and Iranian studies [8, 17]. Our data showed higher ceiling effects than these studies, but our results were similar to those of a previous study in Chinese adolescents [23]. The RE and RP items were changed from two levels in version 1 to five levels in version 2, although the highest scores were still elevated and they ranged from 70.1% to 82.3% but the floor effects were lesser than those in a previous study [5, 8, 17]. Internal consistency reliability was >0.7 for the PF, RP, and RE scales, but the internal reliability of the MH scale was low at 0.37 in our study. Korean people may be free from the influence of two MH items (Calm and peaceful, downhearted and depressed), respectively. These two items were loaded onto a different factor in a previous study on Korean SF-36 [9]. These findings for reliability are comparable with the reliability of 0.34 found in a Chinese study [23]. Factor analysis of individual items produced partial matching of items to their hypothesized components. However, the loading of the items separated into three factors and aggregated into? SF, RE, and RP items. This pattern is unique to the Korean population, as the RE and RP items were also loaded onto the same factor in the Korean SF-36 v2 [9]. Use of item or scale scores rather than use of two summary measures of the SF-12 v2 seems to be more appropriate in the Korean population.

There were some limitations to our present study. Firstly, although we had recruited respondents nationwide, the external validity of the sample would be limited. The age and sex distributions of our sample were similar to those reported in the 2010 national census, but participants in this study reported lower health care utilization than the participants of the 5^th^ KNHANES, which is a national-wide health survey of more than 30,000 people. Lower health care utilization may indicate that our population sample was healthier than the general Korean population. Healthy people may assign a HRQoL score by producing high item scores and a low floor effect. In addition, we did not explore face validity, concurrent validity, test-retest reliability, and responsiveness for health state change. Therefore, further research on the psychometric properties of the SF-12 v2 is needed.

## Conclusions

The Korean SF-12 v2 seems to be a feasible, valid, and reliable instrument for measuring the HRQoL of a general population. The use of scale scores instead of component summaries seems to be more appropriate in Korean people. Further research on other psychometric properties of the Korean SF-12 v2 is desirable.

## Abbreviations

BP: Bodily pain
GH: General health
HRQoL: Health-related quality of life
MCS: Mental component summary
MH: Mental health
PCS: Physical component summary
PF: Physical functioning
RE: Role emotional
RP: Role physical
SF: Social functioning
SF-12 v2: Short form-12 health survey, version 2
VT: Vitality
